# Supplementary material for: Structural basis for topological regulation of Tn3 resolvase
Source: Nucleic Acids Res. 2022 Sep 14;51(3):1001–18. doi: 10.1093/nar/gkac733 (PMC9943657; doi:10.1093/nar/gkac733)
Supplement: gkac733_Supplemental_Files [file gkac733_supplemental_files.zip › List_of_Supplementary_Files.docx]

The following supplementary files are supplied:

- Supplementary Figures
- Tn3synap_twirl.mpg – movie of the Tn3 synaptosome model rotating
- SinSynaptosome.pdb and SinSynaptosome_symmetry.pdb – updated model for the Sin synaptosome. Each file contains one *res* site and the proteins bound to it. The full synaptosome consists of both files. Note that each file also contains the symmetry information necessary to regenerate the other.
- Tn3synaptosome.pdb and Tn3synaptosome_symmetry.pdb – new model for the Tn3 synaptosome. Each file contains one *res* site and the proteins bound to it. The full synaptosome consists of both files. Note that each file also contains the symmetry information necessary to regenerate the other.
