# Supplementary material for: Structural basis for topological regulation of Tn3 resolvase
Source: Nucleic Acids Res. 2022 Sep 14;51(3):1001–18. doi: 10.1093/nar/gkac733 (PMC9943657; doi:10.1093/nar/gkac733)
Supplement: gkac733_Supplemental_Files [file gkac733_supplemental_files.zip › SupplementaryFigures_Aug1_refsrenum.pdf]

Supplementary Figure S1

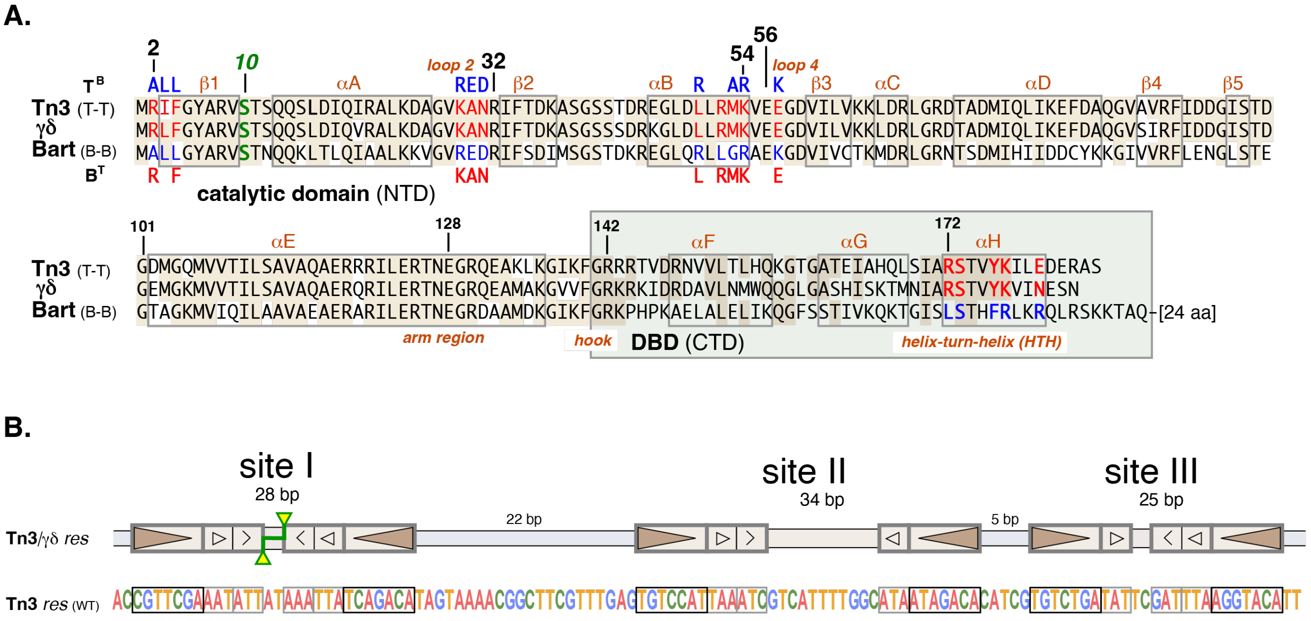

Supplementary Figure S1. Relevant Sequences.

a) Protein sequences. The Tn3 and γδ resolvases are 80% identical; Tn3 and Bart are only 49% identical (shaded residues). Bart has distinct *R* interface and DNA binding (DBD) specificities, and differs at relevant residues (highlighted, red/blue). The catalytic serine (S10), canonical *R* interface residues (2, 32, 54, 56), and E128 in helix E, are also highlighted. The chimaeric catalytic domains designated T^B (Tn3 domain, Bart-type *R* interface) and B^T (Bart domain, Tn3-type *R* interface) have 'patch' mutations at the 10 positions shown, as described (26). The 24-residue CTD tail of the natural Bart resolvase was deleted for all experiments described here.

b) Sequence and organization of the wild-type Tn3 *res* site. The filled triangles denote regions bound by the helix-turn-helix DBD, smaller open triangles denote regions bound by the AT hook, and arrow heads denote regions bound by the C-terminal portion of helix E. Yellow triangles at site I indicate DNA cleavage sites.

Supplementary Figure S2

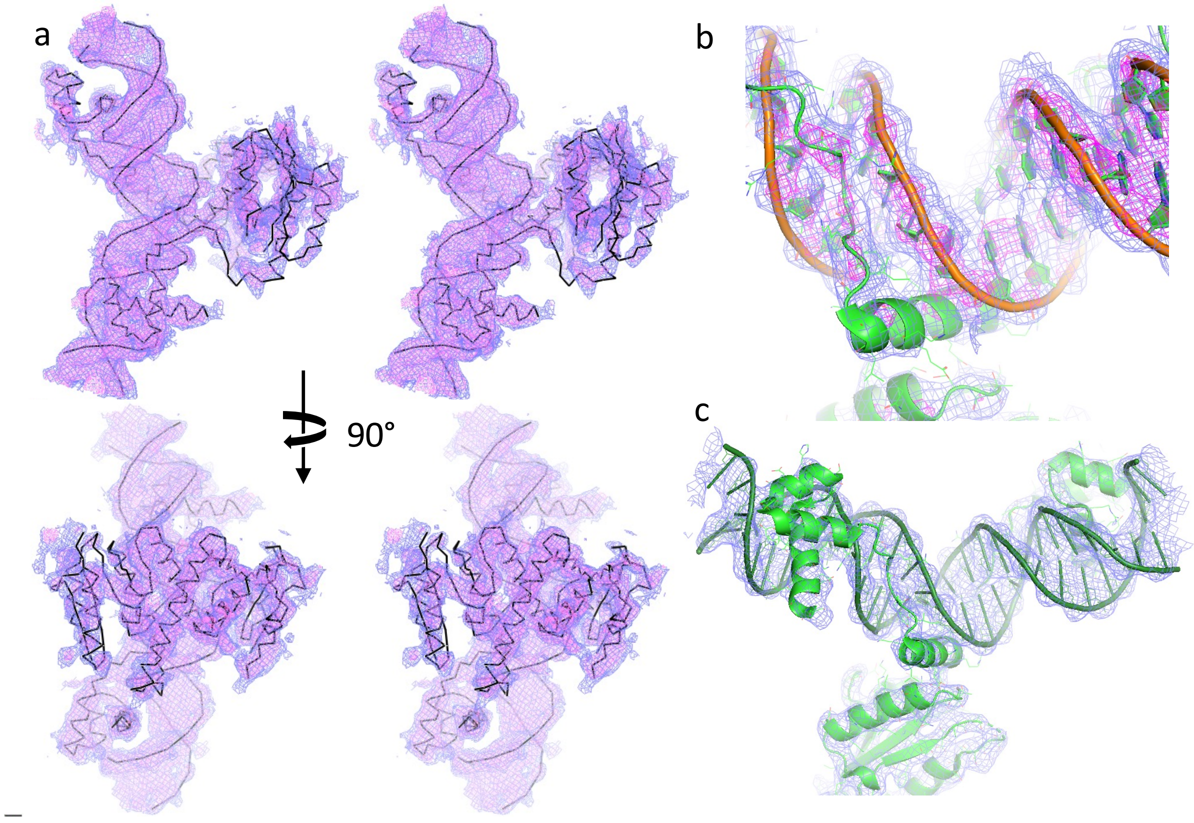

Supplementary Figure S2. Electron density.

a) Experimentally phased electron density map for crystal Form I. Two orthogonal stereo (wall-eyed) views were displayed in PyMOL and contoured at 1 sigma (blue) and 1.5 sigma (pink), with a “carve” radius of 4 Å to remove density for symmetry-related complexes.

b) Final weighted 2Fo-Fc map for Form I, contoured at 1 and 3 sigma, with carve=4 Å.

c) Final weighted 2Fo-Fc map for Form II, countered at 1.5 sigma, with carve = 4 Å.

Supplementary Figure S3

a

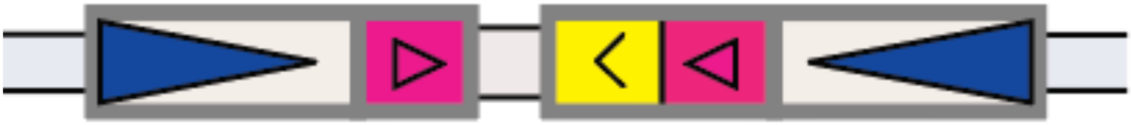

b

TGTCTGATATTCGATTTAAGGTACA

c

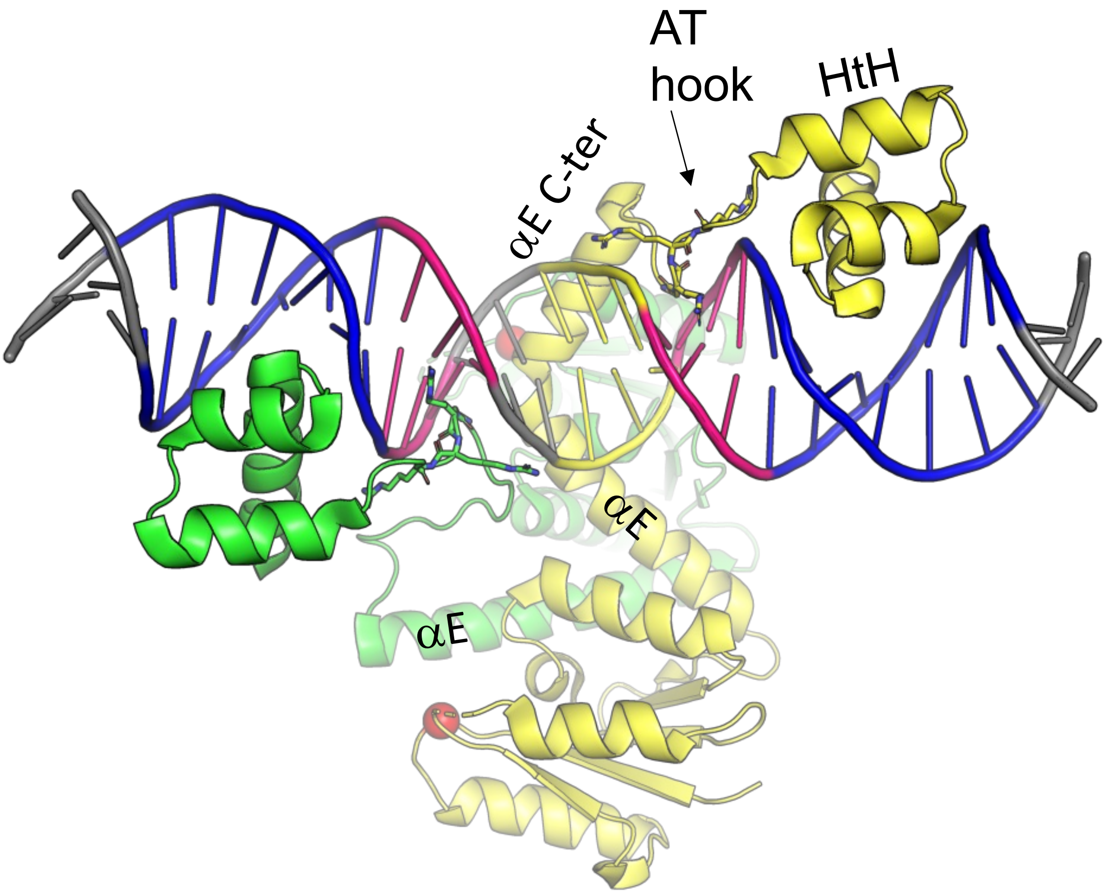

Supplementary Figure S3. Sequence-specific interactions of Tn3 resolvase.

a) Schematic of site III, cartooned with the same symbols as in Figures S1 and S7, but colored as in parts b and c.

b) Sequence of Tn3 site III

c) Tn3 resolvase – site III structure, with the protein colored as in Figures 2 and 3, and the DNA colored as in parts a and b. The AT hook motifs (here GRRR) are shown as sticks. The helix-turn-helix DNA binding domain binds the blue motif; the AT hook binds the pink motif, and the C-terminal segment of helix E binds the yellow motif.

Supplementary Figure S4

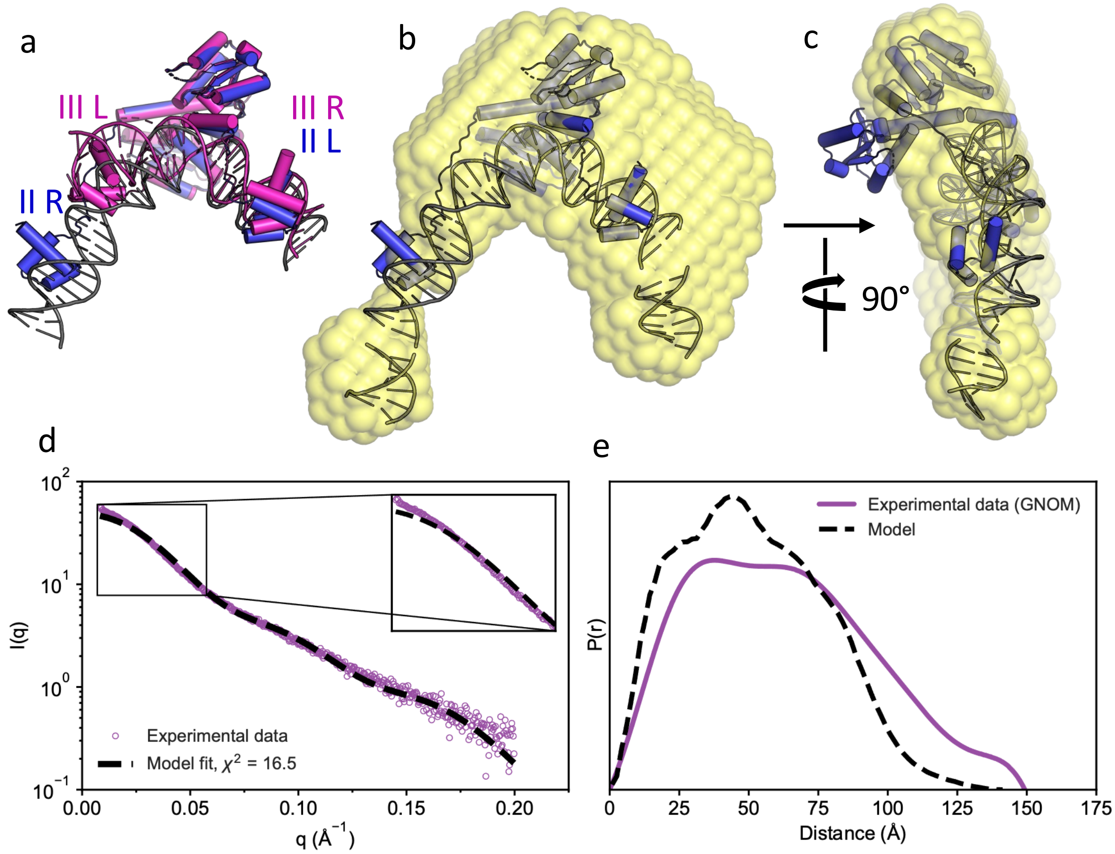

Supplementary Figure S4. Modeling the Tn3 resolvase - site II complex.

a) The site II – bound model (blue and gray) is superimposed onto the site III-bound structure (pink). The catalytic domains were aligned such that site IIIR and site IIL are superimposed.
b) A surface representation of the SAXS bead model is shown in transparent yellow, manually superimposed onto the site II-bound model (blue and gray). Adjacent DNA segments from the synaptosome model are also shown so that the DNA duplex displayed is exactly the same length as that used in the SAXS experiment.
c) Orthogonal view of part (b).
d) The calculated SAXS profile from the atomic model shown in **(b)** and **(c)** (black dashed line) as fit to the experimental data (purple circles). Inset shows a zoom of the low-q region of the curves. The goodness-of-fit between the calculated and experimental curves is 16.5. Not shown: Adding the C-terminal His_6 tags (not visible in the crystal structures) lowers χ^2 to 13.3.
e) The inter-atomic distance distribution calculated explicitly from the atomic model shown in **(b)** and **(c)** (black dashed line), and from the experimental data (purple line) using GNOM. Both distributions have been scaled to unity area.

Supplementary Figure S5

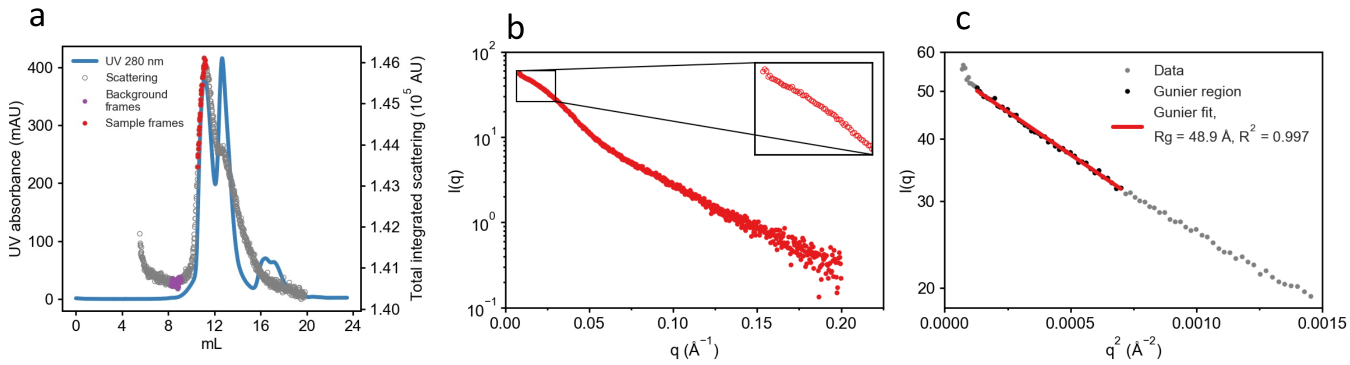

Supplementary Figure S5. SEC-SAXS data for the Tn3 resolvase - site II complex. SAXS data were collected in-line following FPLC-driven size exclusion chromatography of a mixture of the Tn3 resolvase protein and a site II DNA fragment.

a) Post-column FPLC UV A_{280} detector data (blue line) overlaid with the total integrated scattering from each SAXS exposure (circles). The 50 exposures marked as purple circles were merged for use as the background/buffer scattering reference and subtracted from the 40 exposures in red circles from the leading edge of the earliest-eluting peak. These buffer-subtracted data were then scaled and averaged to yield the final SAXS curve.

b) The scaled/merged SAXS curve for the Tn3 resolvase - site II complex. The inset shows a magnification of the low q region. Data were truncated to q < 0.2 Å^{-1}.

c) Guinier fit and radius of gyration (Rg) determination. The SAXS data in the region between the first data point and q = 0.039 are displayed as points, transformed as a Guinier plot (log intensity as a function of q^2). The Guinier region identified by AutoRG is given as black points, with the linear Guinier fit as a red line. The calculated Rg is 48.9 Å.

In all cases, the scattering vector q is given as $4\pi \sin \theta/\lambda$ (Å^{-1}).

Supplementary Figure S6

pMS178 TOP Strand

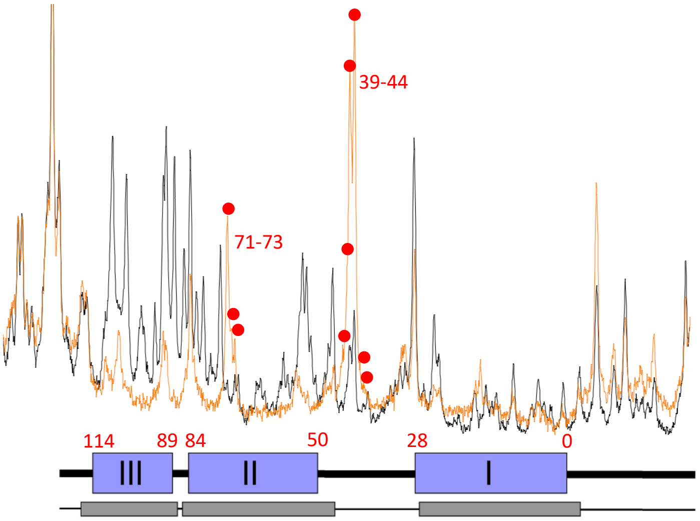

pALY25 Bottom Strand

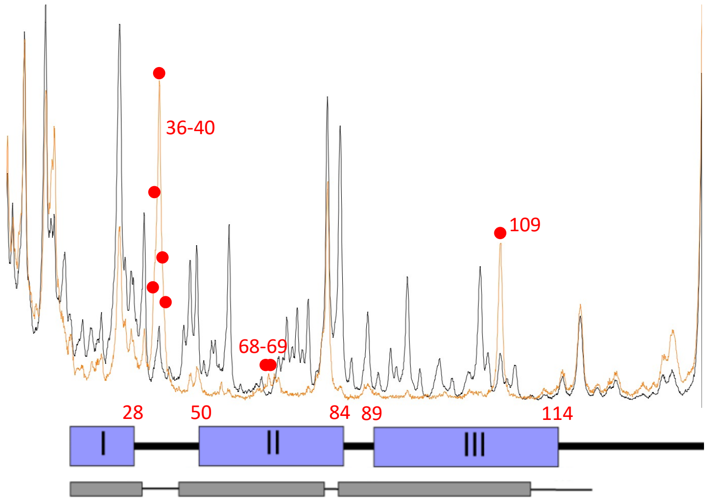

Supplementary Figure S6. DNase I footprinting of the Tn3 synaptosome.
The plots show representative data sets of DNase I strand cleavage intensity within *res*, for naked DNA (black lines) and DNA within the crosslinked synaptosome (orange lines). The positions on the x-axis corresponding to *res* binding sites are indicated by the blue boxes below the plots, and the thinner grey bars indicate regions of general protection from DNase I cleavage by resolvase in the synaptosome. Note that protection of the site I DNA is less complete than for sites II and III; we think that this is due to partial dissociation of site I-bound resolvase subunits during crosslinking of the synaptosomes prior to DNase I treatment ((39); see Materials and Methods section). The small red-filled circles indicate major enhancements of cleavage of the synaptosome DNA by DNase I at specific phosphodiester bonds.

Supplementary Figure S7

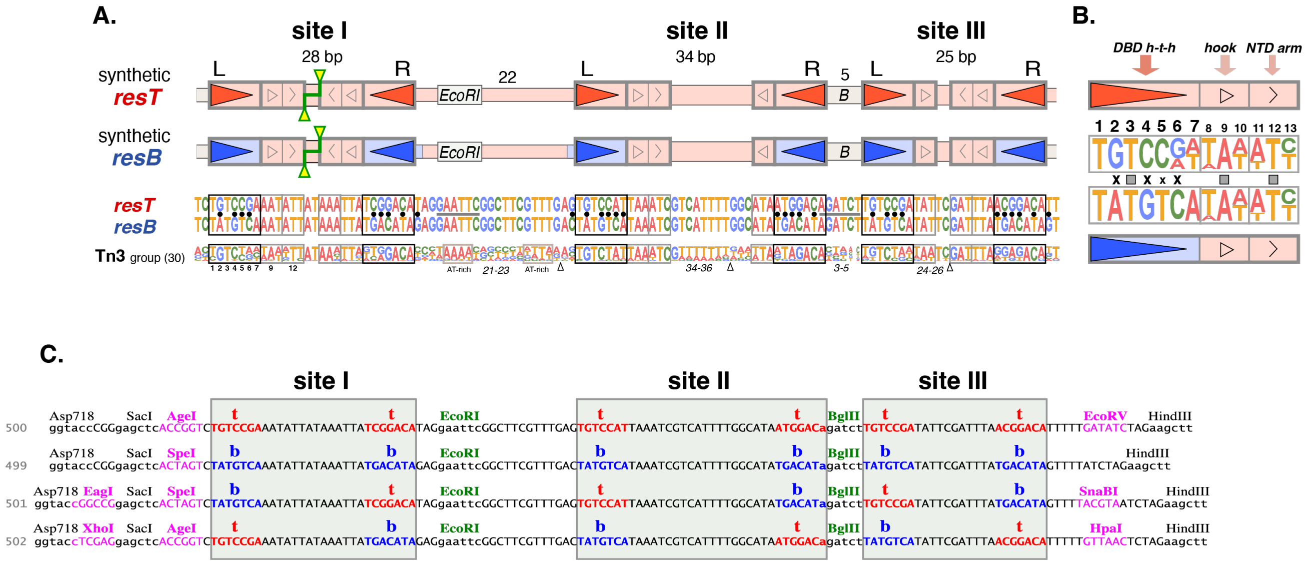

Supplementary Figure S7. Design and sequence of synthetic *res* sites

a) Design of synthetic *res* sites used for targeting experiments. The novel chimaeric *res* sites used here are all hybrids of two synthetic sequences, *resT* and *resB* (cognate sites for Tn3 and Bart resolvases), previously described (26). Both sites are based on wild-type Tn3 *res* sequences (all segments shown in pale red). They differ in the 7 bp motifs (bold red/blue arrows) recognized by the helix-turn-helix part of the Tn3 or Bart DBD. Differences between *resT* and *resB* are highlighted (•) in the sequences. *resT* and *resB* retain many features of natural *res* sites – as exemplified by the *res* site logo based on 30 diverse Tn3-type sites - notably the highly non-random 'linker' sequences between the DBD motifs. (26)

b) CTD targeting motifs. Sequence logos compare the base frequencies (linear scale) in the six half-sites of *resT* and *resB* (excluding positions 11–13 of site IIR and site IIIL). Positions 2, 4, 5 and 6 are thought to be key determinants for selective DBD binding at the *resT* and *resB* motifs; the natural motifs were altered at ten positions to minimize potential selectivity overlaps (see (26)). Note that the motifs contacted by the catalytic domain arm (the C-terminus of helix E) (positions 11-13, open arrows) and the DBD 'hook' (positions 8-10, triangles) are not thought to differ for the Tn3 and Bart resolvases.

c) Synthetic recombination sites used in substrate constructions. Sequences 500 and 499 correspond to *resT* and *resB*, as previously described (26). Sequences 501 and 502 correspond to the hybrid *res* sites (bt tb tb) and (tb bt bt). All other hybrid *res* sites were derived from these four synthetic sequences. The 7 bp motifs at the left and right ends of sites I, II and III are highlighted *red* (Tn3) and *blue* (Bart); within sites I, II and III, there are no further differences between these four sequences. Sites I and II are separated by an *Eco*RI restriction site, and sites II and III by a *Bgl*II site. Restriction sites in *magenta* were used as markers during plasmid construction. Substrate sequences are available on request.

Supplementary Figure S8

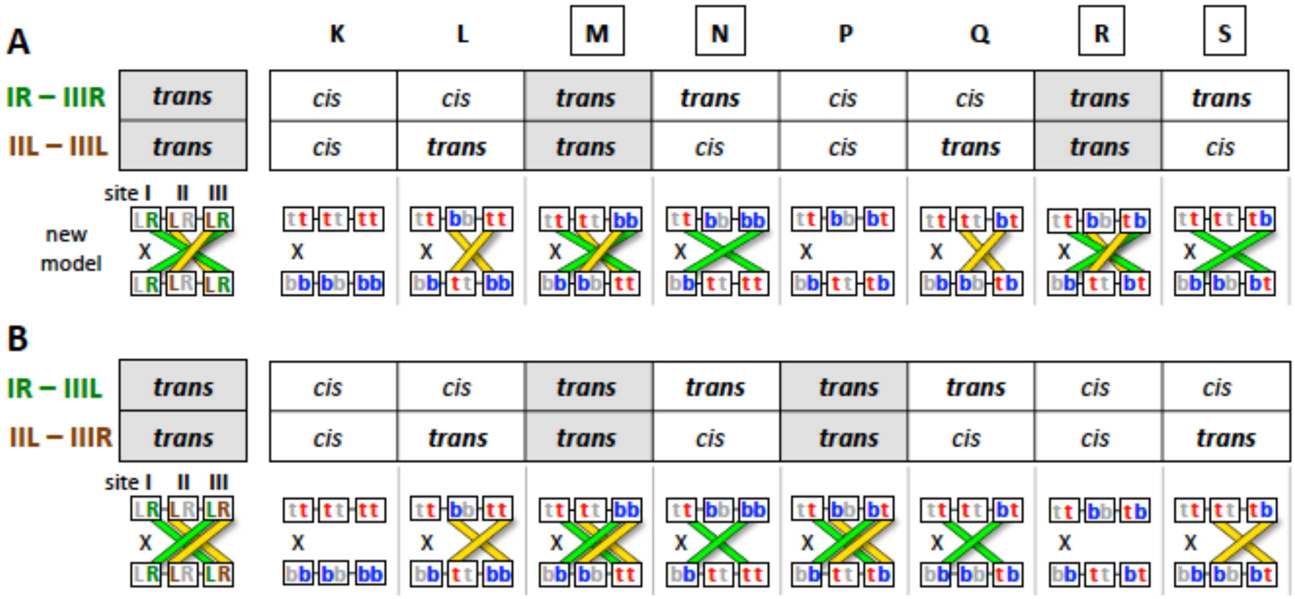

Supplementary Figure S8. Further explanation of how the experiments presented in Figure 7, and the eight recombination substrates used, provide strong support for the new synapse model (a), and rule out alternative arrangements of the dimer-dimer *R* interfaces. Specifically, as shown here, the data rule out an alternative model in which the orientation and connectivity of the site III dimer are reversed (b). Similar arguments (not shown) help to rule out the six other possible permutations of *cis* or *trans* connections between subunits IR + IIIL and IIL + IIIR, or IR + IIIR and IIL + IIIL, including the two arrangements suggested by Sarkis et al. (12), with subunits IIL + IIIL paired *in cis.*

a) This part is identical to Figure 7b, c and d, and shows, for each substrate, whether IR-IIIR and IIL-IIIL interactions between matching subunits can occur *in cis* or *in trans*. The cartoons show, for each substrate, only the half-sites that match *in trans*: IR-IIIR (*green*) and IIL-IIIL (*yellow*). The four substrates that are efficiently recombined, as shown in Figure 7a, are M, N, R and S (boxed).
b) An alternative scenario in which the site III dimer is in the opposite orientation and the interactions are IR-IIIL and IIL-IIIR (both *in trans*). Here, the predicted requirements in order for half-sites to match *in trans* do not correlate with the experimental data.

Supplementary Figure S9

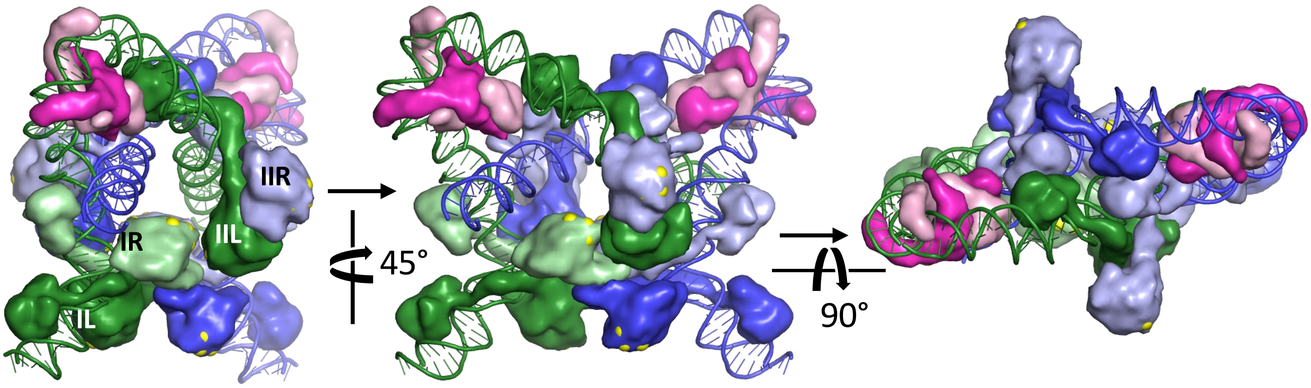

Supplementary Figure S9. Updated model for the Sin Synaptosome.
Three views of the model are shown, in orientations similar to those shown for the Tn3 synaptosome in the main Figure 5. This model differs from that previously published (8) primarily in that the site I-bound subunits are based on a constitutively active Sin rather than γδ resolvase tetramer (see Materials and Methods). Proteins are shown as smoothed surfaces, IHF heterodimers are in pink, and Sin subunits are colored according to the DNA segment they are bound to, with those bound to the right half of each site in a lighter shade than those bound to the left. In the left panel, each protein is labeled according to which half site it is bound to. Also in the left panel, the Cα atoms of *R* interface residues (F52, R54 and D57) are shown as large yellow balls poking out of the protein surface. This model, based on rigid-body docking of substructures, does not perfectly recapitulate the *R* interface between the site I-right and site II-left bound proteins. However, relatively modest adjustments, mostly at the kinked points in the site II-bound proteins' E-helices, would make it do so. Note that in the Sin case, the central node is held together by contacts between the DBDs of the site II-bound proteins (most obvious in the right panel).
